# Adrenomedullin in inflammatory process associated with experimental pulmonary fibrosis

**DOI:** 10.1186/1465-9921-12-41

**Published:** 2011-04-08

**Authors:** Rosanna Di Paola, Elena Talero, Maria Galuppo, Emanuela Mazzon, Placido Bramanti, Virginia Motilva, Salvatore Cuzzocrea

**Affiliations:** 1 IRCCS Centro Neurolesi "Bonino-Pulejo", S.S. 113 Via Palermo, CTR Casazza, Messina, Italy; 2 Department of Clinical and Experimental Medicine and Pharmacology, School of Medicine, University of Messina, Via C. Valeria - Gazzi - 98100 Messina, Italy; 3 Deparment of Pharmacology, School of Pharmacy, University of Seville, 41012 Seville, Spain

## Abstract

**Background:**

Adrenomedullin (AM), a 52-amino acid ringed-structure peptide with C-terminal amidation, was originally isolated from human pheochromocytoma. AM are widely distributed in various tissues and acts as a local vasoactive hormone in various conditions.

**Methods:**

In the present study, we investigated the efficacy of AM on the animal model of bleomycin (BLM)-induced lung injury. Mice were subjected to intratracheal administration of BLM and were assigned to receive AM daily by an intraperitoneal injection of 200 ngr/kg.

**Results and Discussion:**

Myeloperoxidase activity, lung histology, immunohistochemical analyses for cytokines and adhesion molecules expression, inducible nitric oxide synthase (iNOS), nitrotyrosine, and poly (ADP-ribose) polymerase (PARP) were performed one week after fibrosis induction. Lung histology and transforming growth factor beta (TGF-β) were performed 14 and 21 days after treatments. After bleomycin administration, AM-treated mice exhibited a reduced degree of lung damage and inflammation compared with BLM-treated mice, as shown by the reduction of (1) myeloperoxidase activity (MPO), (2) cytokines and adhesion molecules expression, (3) nitric oxide synthase expression, (4) the nitration of tyrosine residues, (5) poly (ADP-ribose) (PAR) formation, a product of the nuclear enzyme poly (ADP-ribose) polymerase (PARP) (6) transforming growth factor beta (TGF-β) (7)and the degree of lung injury.

**Conclusions:**

Our results indicate that AM administration is able to prevent bleomycin induced lung injury through the down regulation of proinflammatory factors.

## Background

Idiopathic pulmonary fibrosis (IPF) is one of the most common forms of interstitial lung disease (ILD) characterized by inexorable, progressive fibrosis involving this critical space. IPF has chronic progressive course, elusive Pathophysiology, no effective treatment options (other than organ transplantation), and is uniformly fatal [1].

The term "idiopathic" suggests there are no known causes for IPF. However, an environmental aetiology for IPF is supported by evidence from several sources [2]. The role of inflammation in the pulmonary fibrosis is still debated, even if several data suggest that the inflammation plays a pivotal role in the genesis of this pathology.

Several studies suggest that fibrosis is the end result of chronic inflammatory reactions induced by a variety of stimuli including persistent infections, autoimmune reactions, allergic responses, chemical insults, radiation[3] and tissue injury [4]. Perivascular inflammatory cell infiltrates are found in lungs from patients with pulmonary hypertension (PH), compared to healthy controls. Patients with idiopathic or associated PH exhibit higher circulating levels and pulmonary expression of various inflammatory cytokines and chemokines, including interleukin-1beta (IL-1β), IL-6 and monocyte chemoattractant protein (MCP-1) [5].

Studies on model mouse of bleomycin-induced pulmonary fibrosis reported that an active inflammatory response invariably precedes the fibrotic response and that fibrogenesis is strictly connected to the development of a response mediated by T CD4+ Th1 type cells [6].

Adrenomedullin (AM) was first isolated by Kitamura et al. from a human pheochromocytoma in 1993 [7]. It is a 52-amino-acid peptide, belonging to the calcitonin gene-related peptide family [8]. AM seems to mediate its activities through binding to a complex receptor composed of the calcitonin receptor like-receptor (CRLR) associated with receptor activity modifying proteins (RAMP)-2 and RAMP-3 [9]. As a consequence of widely spread expression of the peptide and its receptors, the peptide participates in the control of central body functions, such as vascular tone regulation, fluid and electrolyte homeostasis or regulation of the reproductive system [8,10]. However, increasing evidence suggests an important role of AM in inflammatory reactions [11]. Most importantly, high expression of this peptide is demonstrated *in vivo *in humans [12] as well as in animals [13] suffering from severe infection. In particular, increased expression is observed in sepsis and septic shock as well as in LPS-exposed animals. In a model of cecal ligation and puncture in rats, the small intestine was identified as an important source of AM release during polymicrobial sepsis [14] and high expression was observed in the lung in endotoxaemia [15] as well as in acute lung injury induced by hypoxia and LPS [16]. Moreover, an anti-inflammatory role of external AM has been previously suggested in animal models of intestinal bowel disease [17]. Altogether, these observations raise the question of whether AM could play a role in the course of the inflammatory process associated with pulmonary fibrosis. Therefore, the purpose of our study has been to analyze the effects of this peptide, administered i.p, in an experimental model of lung injury by BLM.

## Methods

### Animals

Male CD-1 **(**CD1(ICR) mice (25-35 g; Harlan Nossan; Italy) were housed in a controlled environment and provided with standard rodent chow and water. Animal care was in compliance with Italian regulations on protection of animals used for experimental and other scientific purpose (D.M. 116192) as well as with the EEC regulations (O.J. of E.C. L 358/1 12/18/1986).

### Experimental groups

Mice were randomized into four experimental groups:

• ***BLM + vehicle group***. Mice received intratracheal instillation of BLM (1 mg/kg), and they were treated i.p. with the vehicle for AM (saline 0.9%w/v, 1 h after BLM instillation, and daily (N = 10).

• ***BLM + AM group***. Identical to the BLM + vehicle group but they were administered AM (200 ng/kg i.p.), 1 h after BLM instillation and daily (N = 10).

• ***Sham + vehicle group***. Identical to the BLM + vehicle group but animals received intratracheal instillation of saline (0.9% w/v), instead of BLM, and were treated with vehicle 1 h after saline instillation and daily (N = 10).

• ***Sham + AM group***. Identical to the BLM + AM group but animals received intratracheal instillation of saline (0.9% w/v) instead of BLM, and were treated with AM (200 ng/kg i.p.) 1 h after saline instillation and daily (N = 10).

Mice were killed at 7, 14 or 21 days after BLM instillation for analyses of injury and inflammation. In a separate set of experiments, the same groups were employed.

The dose of adrenomedullin was selected by previous experiments [17].

### Induction of lung injury by bleomycin

Mice received a single intratracheal instillation of saline (0.9% w/v) or saline containing bleomycin sulphate (1 mg/kg body weight) at end-expiration in a volume of 100 μL and the liquid was followed immediately by 300 μL of air, to ensure delivery to the distal airways and were killed after 7, 14 and 21 days by pentobarbitone overdose.

### Measurement of fluid content in lung

The wet lung weight was measured by careful excision of the lung from other adjacent extraneous tissues. The lung was exposed for 48 h at 180°C and the dry weight was measured. Water content was calculated by subtracting dry weight from wet weight.

### Histological examination

Excised lung were taken 7, 14 and 21 days after BLM injection were fixed for 1 week in 10% (w/v) PBS-buffered formaldehyde solution at room temperature, dehydrated, using graded ethanol and embedded in Paraplast (Sherwood Medical, Mahwah, NJ, USA). The sections were prepared and stained by hematoxylin and eosin or by Masson's trichrome stain to identify inflammatory cells, connective tissue and fibrotic lesions. All sections were studied using light microscopy (Dialux 22 Leitz, Zeiss, Milan, Italy). Moreover, the severity of fibrosis was semi-quantitatively assessed, according to the method proposed by Ashcroft and co-workers [18].

### Immunohistochemical localization of TNF-α, IL-1β, ICAM-1, P-selectin, iNOS, nitrotyrosine, PAR and TGF-β

At the end of the experiment, the tissues were fixed in 10% (w/v) PBS-buffered formaldehyde and sections of 8 μm were prepared from paraffin embedded tissues. After deparaffinization, endogenous peroxidase was quenched with 0.3% (v/v) hydrogen peroxide in 60% (v/v) methanol for 30 min. The sections were permeablized with 0.1% (w/v) Triton X-100 in PBS for 20 min. Non-specific adsorption was minimized by incubating the section in 2% (v/v) normal goat serum in PBS for 20 min. Endogenous biotin or avidin binding sites were blocked by sequential incubation for 15 min with biotin and avidin (DBA, Milan, Italy), respectively. Sections were incubated overnight with anti-TNF-α antibody (1:500 in PBS, v/v), anti-IL-1β antibody (1:500 in PBS, v/v), anti-iNOS antibody (1:500 in PBS, v/v), anti-P-selectin antibody (BD Pharmingen, CD62P 1:500), anti-ICAM-1 antibody (BD Pharmingen, CD54, 1:500), anti-nitrotyrosine antibody (1:500 in PBS, v/v), or PAR antibody (1:500 in PBS, v/v) **a**nd anti-TGF-β rabbit polyclonal antibody (1:500 in PBS, v/v). Sections were washed in PBS and incubated with secondary antibody. Specific labeling was detected with a biotin-conjugated goat anti-rabbit or anti-mouse IgG and avidin-biotin peroxidase complex (DBA, Milan, Italy).

### MPO activity

MPO activity, an indicator of polymorphonuclear leukocyte accumulation, was determined as previously described [19] and it was defined as the quantity of enzyme degrading 1 μmol of peroxide min ^-1 ^at 37°C. Results were expressed in U/g wet tissue.

### Measurement of cytokines

Portions of lung were homogenized in PBS containing 2 mmol/L of phenyl-methyl sulfonyl fluoride (Sigma Chemical Co., Milan, Italy) and tissue levels of TNFα and IL-1β were evaluated. The assay was carried out by using a colorimetric, commercial kit (Calbiochem-Novabiochem Corporation, USA) according to the manufacturer instructions. All cytokines determinations were performed in duplicate serial dilutions. Results are expressed as pg/100 g wet tissue.

### Materials

Unless otherwise stated, all compounds were obtained from Sigma-Aldrich Company Ltd. (Poole, Dorset, U.K.). All other chemicals were of the highest commercial grade available. All stock solutions were prepared in non-pyrogenic saline (0.9% NaCl; Baxter, Italy, UK).

### Analysis

All values in the figures and text are expressed as mean ± standard error of the mean (SEM) of N observations. For the in vivo studies, N represents the number of animals studied. In the experiments involving histology or immunohistochemistry, the figures shown are representative of at least three experiments (histological or immunohistochemistry coloration) performed on different experimental days on the tissues section collected from all the animals in each group. Data sets were examined by one- or two-way analysis of variance, and individual group means were then compared with Student's unpaired t-test. A P-value of less than 0.05 was considered significant.

## Results

### Effects of AM on BLM-induced lung injury, body weight, and fluid content

**Figure 1:**
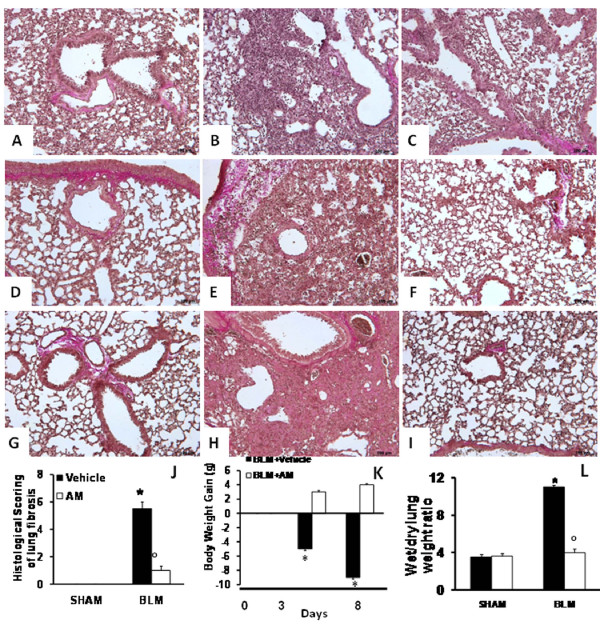
**Effects of adrenomedullin (AM) on bleomycin (BLM)-induced lung injury, body weight, and fluid content**. Masson's trichrome staining of lung sections revealed significant tissue damage (B), when compared with sham-operated animals (A). AM administration caused a decrease of pulmonary lesion, consisting of moderate inflammation and slight fibrosis (C). The histological scoring of fibrosis severity in the lung samples showed in BLM-administered mice a severe degree of injury in relation to sham-operated mice (D); however, AM treatment significantly reduced the lung injury (D). BLM administration was associated with a marked loss in body weight (E), while AM significantly attenuated this weight loss (E). Moreover, BLM administration caused an increase of wet/dry lung weight ratio, when compared with sham-operated mice (F). On the contrary, AM significantly reduced this parameter (F). At 14 and 21 days after treatments, lung sections were subjected to Masson-trichrome staining for the presence of an intense fibrosis. This stain shows collagen in purple. Microphotographs of sections from (D and G) sham-operated animals, (E and H) BLEO (bleomycin-treated mice), and (F and I) AM treated animals show that abundant extracellular matrix (ECM) deposition, alveolar thickening, and severe distortion of lung structures observable in lung sections from BLEO was substantially reduced in AM-treated mice. Figures are representative of at least 3 experiments performed on different experimental days. Data are expressed as mean ± standard deviation from n = 10 mice for each group. *P < 0.01 vs. sham, °P < 0.01 vs. bleomycin + vehicle.

7 days after BLM administration the pulmonary lesions observed in mice consisted of multifocal areas of severe inflammation and intense fibrosis (Figure 1B). Masson-trichrome staining confirmed the presence of an intense fibrosis in the inflammatory focal areas (Figure 1B) when compared with sham-operated animals (Figure 1A). In contrast, a reduced intensity Masson-trichrome staining in AM-treated mice revealed a less severe pattern of pulmonary lesion, consisting of multifocal areas of moderate inflammation and slight fibrosis (Figure 1C). Furthermore, the histological scoring of fibrosis severity in the lung samples showed that the degree of injury is higher in BLM-administrated mice than in AM-treated animals, when compared with sham-operated mice (Figure 1J). The severe lung injury caused by bleomycin administration was associated with a significant loss in body weight, while AM treatment significantly attenuated the loss in body weight (Figure 1K). BLM administration also caused an increase of wet/dry lung weight ratio, due to infiltration of inflammatory cells and edema, in relation to sham-operated mice. On the contrary, AM showed a significant decrease of wet/dry lung weight ratio (Figure 1L).

Moreover, histologic examination of the mice lungs revealed: the abundant extracellular matrix (ECM) deposition and abundant tissue damage in the lungs of BLEO mice after 14 (Figures 1E) and 21 (Figures 1H) days of bleomycin treatment, when compared with sham-operated mice at 14 (Figure 1D) and 21 days (Figure 1G). AM-treatment prevented both ECM deposition and tissue damage at 14 (Figures 1F) and 21 days (Figure 1I).

### Effects of AM on production and expression of TNF-α and IL-1β

**Figure 2:**
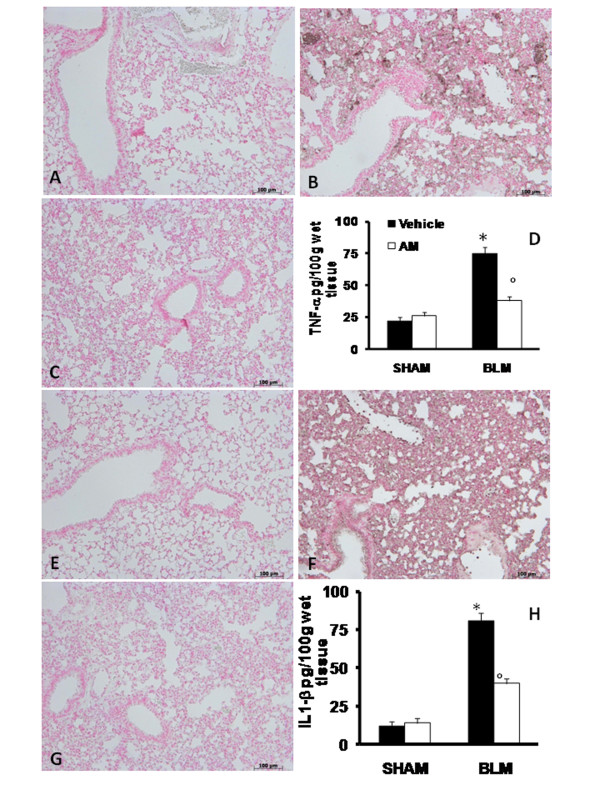
**Effects of adrenomedullin (AM) on production and expression of TNF-α and IL-1β**. The evaluation of the lung production of the pro-inflammatory cytokines TNF-α and IL-1β showed that in samples taken from mice 7 days after bleomycin administration there was a substantial increase in TNF-α (D) and IL-1β (H) formation when compared with sham-operated animals. In contrast, in BLM mice, which had been treated with AM there was a significant inhibition of TNF-α (D) and IL-1β and (H). Immunohistochemical localization of proinflammatory cytokines in lung sections obtained from BLM-treated animals showed positive staining for TNF-α (B) and IL-1β (F); however, in mice treated with AM, the staining for TNF-α (C) and IL-1β (G) was significantly reduced. No positive staining for these cytokines was observed in lung tissues obtained from sham group (A and E, respectively). The figure is representative of at least three experiments performed on different experimental days. Data are expressed as mean ± standard deviation from n = 10 mice for each group. *P < 0.01 vs sham, °P < 0.01 vs bleomycin + vehicle.

To test whether AM may modulate the inflammatory process through regulation of the secretion of cytokines; we analyzed the lung levels of the pro-inflammatory cytokines TNF-α and IL-1β. A substantial increase in TNF-α and IL-1β formation was observed in lung samples taken from mice 7 days after BLM administration, when compared with sham-operated animals (Figures 2D and 2H, respectively). In contrast, a significant inhibition of these cytokines was detected in BLM-administered animals, which had also received AM (Figures 2D and 2H, respectively). As regards immunohistochemical study, tissue sections obtained from BLM-treated animals demonstrated positive staining for TNF-α (Figure 2B) and IL-1β (Figure 2F) mainly localized in the infiltrated inflammatory cells in damaged tissues. In BLM mice treated with AM, the staining for TNF-α (Figure 2C) and IL-1β (Figure 2G) was significantly reduced in relation to BLM-treated group. In the lungs of sham animals no positive staining was observed for TNF-α (Figure 2A) or IL-1β (Figure 2E).

### Effects of AM on adhesion molecules expression, and MPO activity

**Figure 3:**
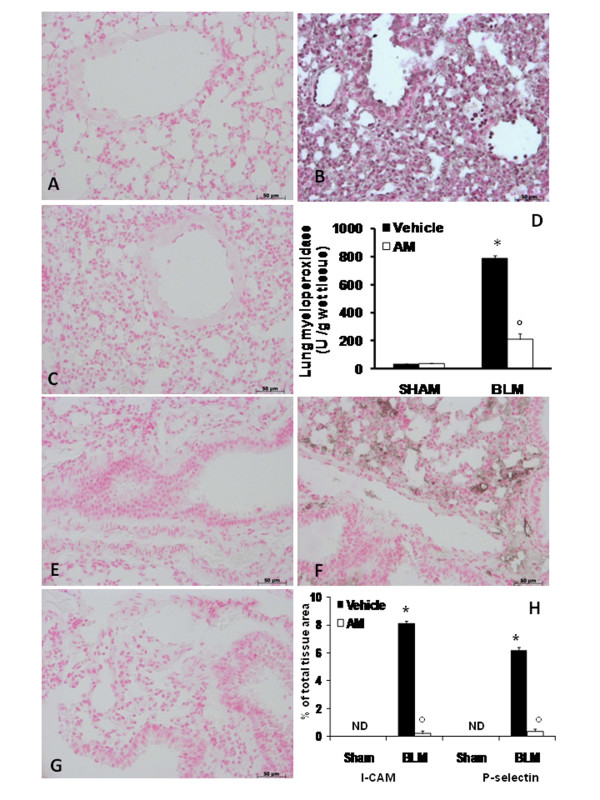
**Effect of adrenomedullin (AM) on adhesion molecules expression and MPOactivity**. Immunohistochemical analysis of lung sections obtained from BLM-treated mice revealed a positive staining for ICAM-1 (B) and P-selectin (F) in the injured tissues, mainly localized around the vessels. In BLM mice treated with AM, the staining for ICAM-1 (C) and P-selectin (G) was significantly reduced when compared with BLM group No positive staining for ICAM-1 and P-selectin was observed in lung tissues obtained from sham-operated mice (A and E, respectively). Densitometry analysis of immunocytochemistry photographs (n = 5 photos from each sample collected from all mice in each experimental group) for ICAM-1 and P-selectin from lung tissues was assessed (H). The assay was carried out by using Optilab Graftek software on a Macintosh personal computer (CPU G3-266). Data are expressed as % of total tissue area. MPO activity was increased by BLM-administration, when compared with lung tissues from sham animals (C). In contrast, a decrease in this enzyme activity was observed in mice treated with AM (C). The figure is representative of at least 3 experiments performed on different experimental days. Data are expressed as mean ± standard deviation from n = 10 mice for each group. *P < 0.01 vs sham, °P < 0.01 vs bleomycin + vehicle.

The severe lung injury caused by BLM administration was associated with the increase of immunohistochemical staining of adhesion molecules, such as ICAM-1 and P-selectin, in the lung sections obtained from BLM-administered mice (Figures 3B and 3F, respectively, see densitometry analysis in Figure 3H). In AM-treated mice, the positive immunostaining for ICAM-1 and P-selectin in the lung (Figures 3C and 3G, respectively, see densitometry analysis in Figure 3H) was significantly reduced. No positive staining for anti-ICAM-1 antibody was observed in lung tissue section of sham-operated mice (Figure 3A, see densitometry analysis in Figure 3H). No positive staining for P-selectin was found in lung tissue section from sham-operated mice (Figure 3E, see densitometry analysis in Figure 3H). Moreover, adhesion molecules expression appeared to be correlated with an influx of leukocytes into the lung tissue. Therefore, we investigated the role of AM on neutrophil infiltration by measurement of MPO activity. Levels of this enzyme activity were increased by BLM administration, when compared with lung tissues obtained from sham animals (Figure 3D). In contrast, a decrease of MPO activity was observed in tissue sections taken from BLM-administered mice and treated with the peptide (Figure 3D).

### Effects of AM on BLM-induced iNOS expression, nitrotyrosine, and PAR formation

**Figure 4:**
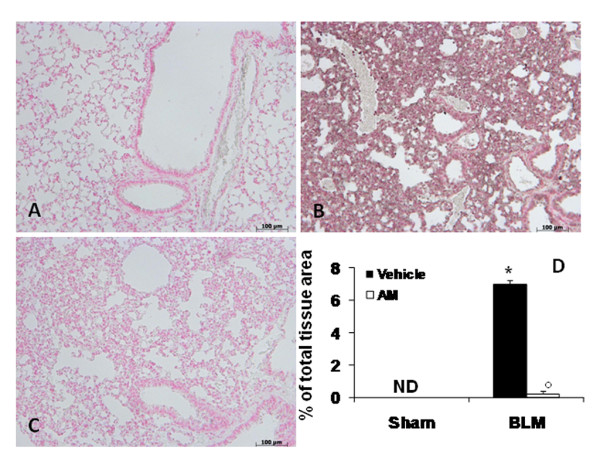
**Effects of adrenomedullin (AM) on bleomycin (BLM)-induced iNOS expression**. Immunohistochemical localization for iNOS revealed a positive staining for this enzyme in lung sections obtained from BLM-treated mice (B). In BLM mice treated with AM, the staining for iNOS (C) was significantly reduced when compared with BLM mice. No positive staining for iNOS was observed in lung tissues obtained from sham-operated mice (A). Densitometry analysis of immunohistochemistry photographs (n = 5 photos from each sample collected from all mice in each experimental group) for iNOS was assessed (D). The assay was carried out by using AxioVision on a personal computer. The figure is representative of at least three experiments performed on different experimental days. Data are expressed as % of total tissue area and are mean ± standard deviation from n = 10 mice for each group. *P < 0.01 vs sham, °P < 0.01 vs bleomycin+ vehicle.

**Figure 5:**
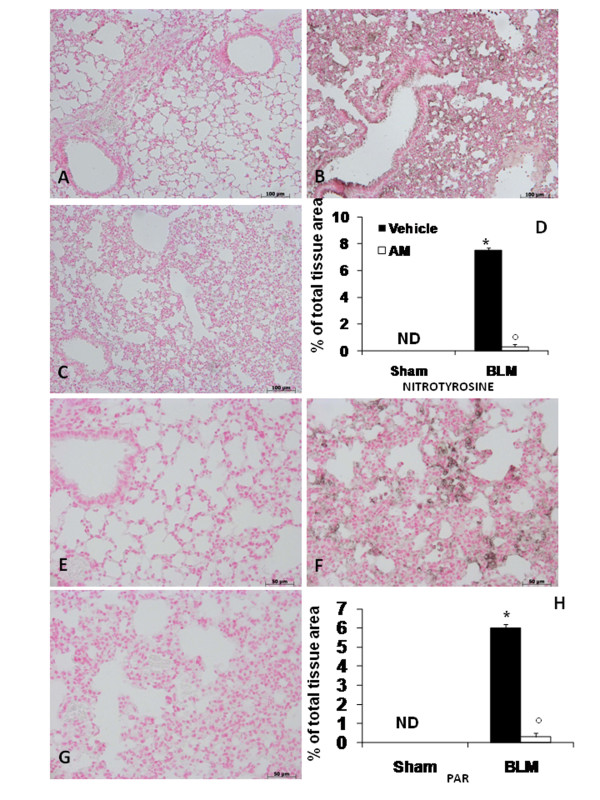
**Effects of adrenomedullin (AM) on bleomycin (BLM)-induced nitrotyrosine and PAR formation**. Immunohistochemical analysis of lung sections obtained from mice treated with BLM revealed positive staining for nitrotyrosine (B). In BLM mice treated with AM, positive staining for nitrotyrosine was significantly reduced (C). Moreover, immunohistochemical analysis of lung sections obtained from mice treated with BLM revealed a positive staining for PAR (F). In contrast, positive staining for PAR was significantly reduced in the lungs of BLM mice treated with AM (G). No positive staining for nitrotyrosine (A) and PAR (E) was observed in lung tissues obtained from sham-operated mice. Densitometry analysis (D and H) of immunohistochemistry photographs (n = 5 photos from each sample collected from all mice in each experimental group) for nitrotyrosine and PAR was assessed. The assay was carried out by using AxioVision on a personal computer. The figure is representative of at least three experiments performed on different experimental days. Data are expressed as % of total tissue area and are mean ± standard deviation from n = 10 mice for each group. *P < 0.01 vs sham, °P < 0.01 vs bleomycin+ vehicle.

iNOS expression was assessed in samples of pulmonary tissue by immunohistochemistry analysis. Our results showed no positive staining for this enzyme in the lung tissues obtained from sham animals (Figure 4A, see densitometry analysis in Figure 4D). On the contrary, lung sections obtained from BLM-treated mice revealed positive staining for iNOS (Figure 4B, see densitometry analysis in Figure 4D), while no immunostaining for iNOS was found in the lungs of BLM-treated mice that had been treated with AM (Figure 4C, see densitometry analysis in Figure 4D). Immunohistochemical analysis of lung sections obtained from mice treated with BLM also revealed positive staining for nitrotyrosine (Figure 5B, see densitometry analysis in Figure 5D). In BLM mice treated with AM, positive staining for nitrotyrosine was significantly reduced (Figure 5C, see densitometry analysis in Figure 5D). Moreover, immunohistochemical analysis of lung sections obtained from mice treated with BLM revealed a positive staining for PAR (Figure 5F, see densitometry analysis in Figure 5H). In contrast, no staining for PAR was found in the lungs of BLM mice treated with AM (Figure 5G, see densitometry analysis in Figure 5H). There was no staining for either nitrotyrosine or PAR in lungs obtained from sham group (Figure 5A and 5E, respectively, see densitometry analysis in Figures 5D and 5H).

### Effects of AM on BLM-induced TGF-β

**Figure 6:**
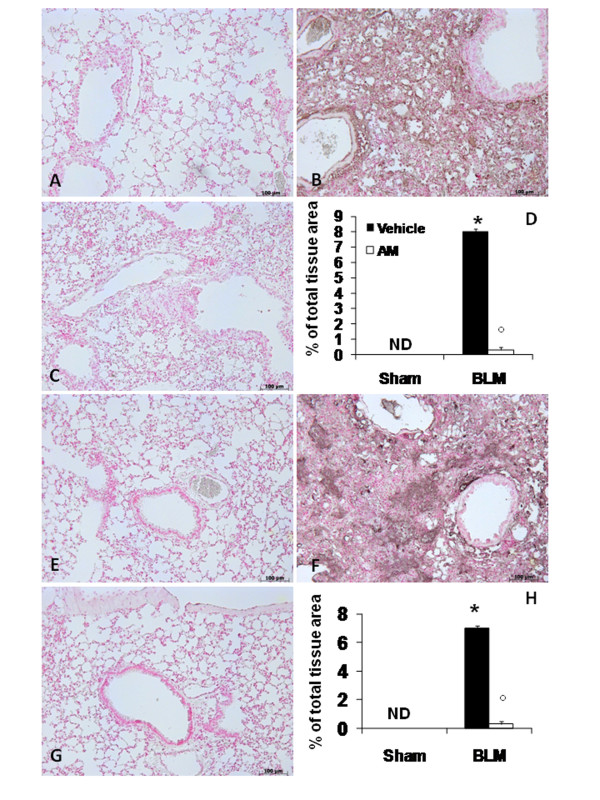
**Effects of adrenomedullin (AM) on TGF-β**. immunohistochemical analysis of lung sections obtained from mice treated with BLM revealed positive staining for TGF-β at 14 (B) and 21 (F) days after treatments. In BLM mice treated with AM, positive staining for TGF-β was significantly reduced at 14 (C) and 21 (G) days. Densitometry analysis (D and H) of immunohistochemistry photographs (n = 5 photos from each sample collected from all mice in each experimental group) for TGF-β was assessed. The assay was carried out by using AxioVision on a personal computer. The figure is representative of at least three experiments performed on different experimental days. Data are expressed as % of total tissue area and are mean ± standard deviation from n = 10 mice for each group. *P < 0.01 vs sham, °P < 0.01 vs bleomycin+ vehicle.

In advanced idiopathic pulmonary fibrosis, extensive TGF-β deposition can be detected primarily in epithelial cells in areas of lung regeneration and remodelling. Thus, we studied total TGF-β in lung sections by immunohistochemistry. Bleomycin induced a remarkable increase of TGF-β staining in the alveolar epithelium and in the inflammatory infiltrate at 14 (Figure 6B see densitometry analysis D) and 21 days (Figure 6F see densitometry analysis H). In contrast, AM-treated mice did not exhibit such an increase at 14 (Figure 6C see densitometry analysis D) and 21 days (Figure 6G see densitometry analysis H). No alteration was observed in sham-operated mice at 14 (Figure 6A see densitometry analysis D) and 21 days (Figure 6E see densitometry analysis H).

## Discussion

This study examined the beneficial effect of AM on BLM-induced pulmonary fibrosis; in particular, our results indicate that AM has strong anti-inflammatory properties resulting in a reduced: (1) MPO activity, (2) cytokines and adhesion molecules expression, (3) iNOS expression, (4) the nitration of tyrosine residues (5) PAR formation, a product of PARP-1 activity, and (7) the degree of lung injury tissues in mice subjected to BLM instillation. AM can play a master role in orchestrating differential regulation among tissues during inflammation because of its capacity to bind to multiple classes of receptors [20] and elicit different tissue responses in specific tissue sites. In essence, AM is both a hormone and a cytokine [20]. It can simultaneously regulate aspects of regional blood flow, immunological recruitment, and preferential nutrient use by tissues during the inflammatory response. Many of the responses of body tissues to an inflammatory insult are triggered and modulated by cytokines. Most relevant to the topic at hand is the tight relationship between proinflammatory cytokines, like TNF-α and IL-1β, and AM during the onset of systemic as well as localized tissue inflammatory response [21]. BLM model, it has been shown that the cytokine network is capable of modulating the different phases of lung fibrosis pathogenesis [22]. Among the several cytokines and chemokines that have been implicated in the pathogenesis of lung fibrosis, particular relevance has been given to IL-1 and TNF-α.

Recent studies suggest that AM plays a role in the complex network of pulmonary cytokines. In vitro data showed that AM inhibits cytokine-induced neutrophil chemoattractant secretion from lipopolysaccharide-stimulated rat alveolar macrophages, and suppress TNF-α production in IL-1β stimulated Swiss 3T3 cells. An in vivo study demonstrates a significant suppression of pulmonary TGF-β1 and IL-1β mRNA expression by aerosolized AM [23]. In the present study, we confirm that the model of lung injury used leads to a substantial increase in the levels of TNF-α and IL-1 in the lung after BLM administration and we report by first time that the production of the pro-inflammatory cytokines are significantly attenuated by the treatment with AM.

In pulmonary fibrosis, the fibrotic process is thought to be initiated by a variety of events following cell migration including extracellular matrix degradation [24]. An important step in the inflammatory process is the induction of cell adhesion molecules such as intercellular adhesion molecules (ICAM). Strong adhesion between leukocytes and endothelial cells is promoted by ICAM, which can be driven by TNF-α [25].

The identity and role of the adhesion molecules involved in the fibrotic process are unknown. Hamaguchi et al. shown a significant decrease of pulmonary fibrosis in a mouse model lacking ICAM expression suggesting that these adhesion molecules provide a critical role in the development of pulmonary fibrosis [26]. We confirm in the present study that BLM instillation leads to a substantial increase in adhesion molecules expression in the lung. We also report that AM treatment significantly reduced the expression. Thus it is conceivable that AM, by decreasing the expression of TNF-α, which is known to regulate the production of ICAM, leading to a reduction of inflammation and fibrosis accordingly.

There is compelling evidence that endogenous NO plays a key role in physiological regulation of airway functions and is implicated in airway disease. In an inflammatory micro environment NO, and related compounds, are produced by a wide variety of residential and inflammatory cells in the respiratory system [27]. This reaction is catalyzed by iNOS in macrophages and epithelial, endothelial, and vascular smooth-muscle cells. This isoform is regulated at a pre-translational level and can be induced by proinflammatory cytokines, such as TNF-α, and IL-1β. The immunohistochemistry method applied in our study revealed a positive staining of iNOS in lung sections after BLM administration and that AM reduced the staining in these tissues.

In addition, in chronic airway inflammation, inflammatory cells (eosinophils, neutrophils, monocytes and macrophages) may become activated and generate oxidants in response to various stimuli ("oxidative stress") [28]. The univalent reaction of oxygen to superoxide anion (O_2_^-^) is an important step in the formation of oxidants. Exaggerated production of NO, in the presence of "oxidative stress", may produce the formation of strong oxidizing reactive nitrogen species, such as peroxynitrite (ONOO^-^) [27]. Nitrotyrosine formation has been used as a marker of endogenous ONOO^- ^formation [29] although it has been demonstrated that other reactions can also induce tyrosine nitration, e.g. the reaction of nitrite with hypochlorous acid and the reaction of MPO with Hydrogen peroxide (H_2_O_2_), both leads to the formation of nitrotyrosine [30]. Thus, increased nitrotyrosine staining is considered as an indicator of 'increased nitrosative stress' rather than a specific marker of the generation of ONOO^- ^[30]. We have found that nitrotyrosine is indeed present in lung sections after BLM administration and that AM treatment reduced this staining in the tissues. We propose that AM, acting on cytokines, inhibits the iNOS expression, and the subsequent formation of nitric oxide, resulting in the reduction of nitrosative stress.

Overproduction or reactive oxygen and nitrogen intermediates (ROI and RNI, respectively) may cause DNA breakage and can lead to PARP activation. Although PARP activation may enhance the repair of damaged DNA, it may also be deleterious for the cells in severe oxidative stress situations. Excessive ROI/RNI production may cause un-repairable DNA damage leading to the over activation of PARP-1, depletion of NAD^+^, the substrate of PARP-1. Low NAD^+ ^levels slow down glycolysis resulting in suppressed ATP production. Resynthesis of NAD^+ ^also consumes ATP and depletion of these two key energy metabolites leads to cell dysfunction or even cell death [31]. In our study, we also demonstrate that AM treatment reduced the increase in PARP activation in the lung from BLM-treated mice.

Furthermore, AM proved efficacious to significantly lower total and biologically active TGF-β levels. TGF-β plays a central role in fibrotic disorders in different organs, including fibrosis of the lung. In fact, it stimulates collagen and fibronectin production in fibroblasts [32] on the other hand, it can suppress the production of proteases that degrade the extracellular matrix [33]. TGF-β has been shown to be increased in bleomycin-induced lung fibrosis in the alveolar inflammatory infiltrate [34]. Secretion of active TGF-β by alveolar macrophages is augmented after bleomycin administration in mice, whereas latent TGF-β secretion remains elevated for a prolonged length of time, and it is probable that the extent of inflammation and fibrosis in this model depend on the quantity of active TGF-β available [35]. In our study, we demonstrate that AM treatment reduced the TGF-β increase in the lung from BLM-treated mice.

## Conclusion

These data support the hypothesis that AM is an inhibitor of BLM-induced lung fibrosis and this protective effect is observed also by a significant reduction of the oedema formation, tissue damage and reduced content of collagen. Also, the treatment with AM reduced the loss of body weight and improved the survival of the mice. In conclusion, we hypothesize that the anti-inflammatory properties of AM may be related to its ability to decrease the production and expression of proinflammatory cytokines, as our work has demonstrated. This property leads us to imagine the existence of an intricate interaction between AM and cytokines, leading to a modulation of inflammatory process associated with lung fibrosis. It is clear that will require further and detailed studies.

## List of abbreviations

**AM**: Adrenomedullin; **ATP**: Adenosine triphosphate; **BLM**: Bleomycin; **CRLR**: Calcitonin receptor like-receptor; **H**_**2**_**O**_**2**_: Hydrogen peroxide; **ICAM-1**: Inter-Cellular Adhesion Molecule 1; **IL-1β**: Interleukin-1beta; **IL-6**: Interleukin-6; **ILD**: Interstitial lung disease; **iNOS**: Inducible nitric oxide synthase; **IPF**: Idiopathic pulmonary fibrosis; **LPS**: Lipopolysaccharide; **MCP-1**: Monocyte chemoattractant protein; **MPO**: Myeloperoxidase; **NAD**: Nicotinamide Adenine Dinucleotide; **NO**: Nitric oxide; **O**_**2**_^-^: Superoxide anion; **ONOO**^-^: Peroxynitrite; **PAR**: Poly (ADP-ribose); **PARP-1**: Poly(ADP-ribose) polymerase-1; **PBS**: Phosphate buffered saline; **PH**: Pulmonary hypertension; **PMNs**: Polymorphonuclear leukocytes; **(RAMP)-2-3**: Receptor activity modifying proteins; **RNI**: Reactive nitrogen intermediates; **ROI**: Reactive oxygen intermediates; **SEM**: Standard error of the mean; **TNF-**α: Tumor necrosis factor-α.

## Conflict of interests

The authors declare that they have no competing interests.

## Authors' contributions

The work presented here was carried out in collaboration between all authors.

SC defined the research theme.

RDP, MG and EM designed methods and experiments, carried out the laboratory experiments, analyzed the data, interpreted the results and wrote the paper.

ET and VM co-designed the dispersal and colonization experiments, and co-worked on associated data collection and their interpretation.

SC and PB co-designed experiments, discussed analyses, interpretation, and presentation.

All authors have contributed to, seen and approved the manuscript.
